# Acute Limb Ischemia: A Rare Complication of COVID-19

**DOI:** 10.7759/cureus.11488

**Published:** 2020-11-15

**Authors:** Sidra A Hasan, Ayema Haque, Fatima Nazir

**Affiliations:** 1 Anesthesiology and Intensive Care, Shaheed Mohtarma Benazir Bhutto Institute of Trauma, Karachi, PAK; 2 Internal Medicine, Dow University of Health Sciences, Civil Hospital Karachi, Karachi, PAK

**Keywords:** arteriosclerosis obliterans, tocilizumab, thromboembolism, cytokines, covid-19

## Abstract

A 60-year-old male with a history of primary hypertension presented to the emergency department of a tertiary care hospital, in Pakistan, with complaints of fever, cough, and shortness of breath. He tested positive for the severe acute respiratory syndrome coronavirus 2 (SARS-CoV-2) polymerase chain reaction, with bilateral infiltrates found in chest X-ray. At admission, oxygen saturation was 80% on room air; hence, he was immediately put on non-invasive ventilation. Laboratory investigation revealed elevated D-dimer, international normalized ratio, and total leukocyte count. C-reactive protein was markedly elevated (82.5 mg/L), indicating the state of a cytokine release syndrome (CRS). Treatment started with antibiotics, prophylactic enoxaparin (40-mg subcutaneous once daily), methyl prednisone 60 mg BD and multivitamins. Intravenous tocilizumab (TCZ) 6 mg/kg was started from Day 1 to address the CRS. On Day 3, he complained of pain in the right lower limb with signs of hypothermia, numbness, and slight blackening of the right foot. Peripheral pulses were not palpable, and vascular ultrasound showed no vascular flow in the popliteal, anterior and posterior tibial, and dorsalis pedis artery. The Vascular Surgery department declared the limb unsalvageable and right limb above-knee amputation. On Day 9, the right foot was blackened and atrophied extending up to the knee. Above-knee amputation was done, and he was discharged on rivaroxaban after 48 hours of observation. We conclude that heparin is effective in treating coronavirus disease 2019-associated coagulopathy, while TCZ, simultaneously, decreases the severity of CRS. Our case suggests that the concomitant use of TCZ and anticoagulation therapy can be beneficial in patients presenting with arterial and venous thrombosis.

## Introduction

Coronavirus is a positive-sense, single-stranded, enveloped RNA virus with a helical capsid. The 2019 novel coronavirus (2019-nCoV) pandemic or coronavirus disease (COVID-19), initially identified in Wuhan, Hubei, China, in December 2019, has spread exponentially to 200 countries, causing a global pandemic [1]. On January 3, 2020, WHO declared COVID-19 as a public health emergency of international concern (PHEIC) [2].

COVID-19 has a broad spectrum of clinical manifestations, including bilateral pneumonia, acute respiratory distress syndrome (ARDS), endothelial dysfunction, hypercoagulability, and multiorgan failure. Markedly elevated D-dimer levels and fibrin degradation products coupled with the overproduction of cytokines (interleukin [IL]-2, IL-6, IL-7, IL-10, granulocyte colony stimulating factor [G-CSF], interferon-γ inducible protein 10 kD [IP-10], monocyte chemoattractant protein 1 [MCP1], macrophage inflammatory protein 1-α [MIP1-α], and tumor necrosis factor-α [TNF-α]) lead to an increased risk of microthrombosis, vascular hyperpermeability, disseminated intravascular coagulation (DIC), and multiorgan failure [1,3]. COVID-19-associated coagulopathy (CAC) includes both arterial and venous thromboembolism (VTE) [4]. Acute limb ischemia (ALI) is a vascular emergency. The increasing incidence of ALI in COVID-19 patients has been noted [5]. COVID-19 patients presenting with acute lower extremity ischemia due to arteriosclerosis obliterans, and venous thrombosis simultaneously, are linked to poor prognosis and increased mortality [1].

Tocilizumab (TCZ) is an IL-6 inhibitor that has shown favorable results in COVID-19 as demonstrated through multiple ongoing trials [6]. Anticoagulation therapy, especially with lower molecular weight heparin (LMWH), is associated with better prognosis in patients with CAC [7]. We report a case of a 60-year-old male with COVID-19, who presented with fever and pneumonia and later developed complications of cytokine release syndrome (CRS), venous thrombosis, and ALI. Our case highlights the importance of the timely use of TCZ and heparin therapy in managing CAC for improved outcomes.

## Case presentation

**Figure 1:**
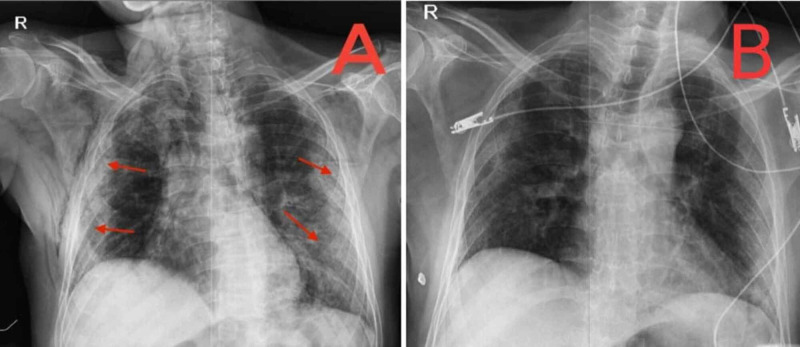
Chest X-ray of the patient Chest X-ray showing progression of bilateral peripheral and basal lung infiltrates on (A) the day of admission and (B) the day before discharge.

A 60-year-old male came to the emergency department of a tertiary care hospital, in Pakistan, with complaints of fever for one week, cough, and shortness of breath for two days. He was tested positive for severe acute respiratory syndrome coronavirus 2 (SARS-CoV-2) before admission. The polymerase chain reaction (PCR) test came positive and chest X-ray revealed bilateral infiltrates as shown in Figure 1A. The patient was diagnosed with severe COVID-19 infection and was admitted to our dedicated COVID-19 ICU. His past medical history included essential hypertension for the past 10 years, which was in control by his medication.

Examination at the time of admissions showed a regular heart rate of 100 beats per minute, non-invasive blood pressure (NIBP) of 155/88 mmHg, temperature 37.5°C, respiratory rate of 30 breaths per minute, and pulse oximeter showed 80% saturation on room air. The patient was immediately given non-invasive 100% FiO_2_, which improved saturation to 95%. Continuous monitoring of vitals was done using the Life Scope VS BSM-3000 series monitor (Nihon Kohden, Tokyo, Japan) for NIBP measurement, pulse oximetry, and electrocardiogram.

Initial lab investigations showed an elevated white blood cell (WBC) count of 13.9×10^9^/L, elevated neutrophil percentage of 91%, decreased lymphocyte percentage of 6%, and a decreased platelet count of 95×10^9^/L. The biochemical examination displayed normal serum creatinine 0.7 mg/dL, slightly elevated blood urea nitrogen 25 mg/dL, serum lactate dehydrogenase 1397 U/L. C-reactive protein (CRP) was markedly increased with a level of 82.5 mg/L, and procalcitonin was normal at 0.3 ng/mL. Coagulation function displayed an elevated D-dimer level of 1.96 mg/L, normal prothrombin time of 14.2 seconds, elevated international normalized ratio of 1.35, and elevated activated partial thromboplastin time of 28.4 seconds on Day 1. Arterial blood gases showed a slightly alkalotic pH of 7.48, decreased pCO_2_ of 27 mmHg, decreased pO_2_ of 60.7 mmHg, and increased lactate level of 3.2 mmol/L. His laboratory findings are shown in Table 1. However, one of the limitations to this study is that his complete cytokine assay was not performed.

**Table 1:** Laboratory parameters on Days 1, 2, 3, 13, and 14 CRP, C-reactive protein. Day 13 was the pre-operative day and Day 14 was postoperative. A drastic decline in CRP levels can be seen on Day 2 after the initiation of tocilizumab therapy.

	Reference range	Day 1	Day 2	Day 3	Day 13 (pre-operative)	Day 14 (postoperative)
Total leukocyte count (×10^9^/L)	4-11	13.9	14.7	15.0	9	8.6
Neutrophil percentage	50-75	91	89	78	62	67
Lymphocyte percentage	20-50	6	4	15	30	28
Platelet count (×10^9^/L)	150-400	95	150	200	252	236
Hemoglobin (g/dL)	13-18	14.2	15.6	14.3	13	12.6
CRP (mg/L)	<5	85	8	0.4	0.5	2
D-dimer (mg/L)	<0.5	1.96	1.55	1.38	0.3	0.3
Prothrombin time (seconds)	26	14.2	11	12.1	12	11.9
International normalized ratio	1.05	1.35	1.05	1.15	1.1	1.09
Activated partial thromboplastin time (seconds)	26	28.4	23.8	25.2	26	25

The patient's treatment plan included antibiotics, prophylactic enoxaparin, 40 mg subcutaneous once daily, symptomatic, and supportive treatment, including steroids and vitamins. The patient was also found to have CRS by aggravated inflammatory markers, for example, CRP levels [8]. Intravenous TCZ 6 mg per kg was given on the day of admission. Oral feed along with oral and intravenous hydration was continued.

**Figure 2:**
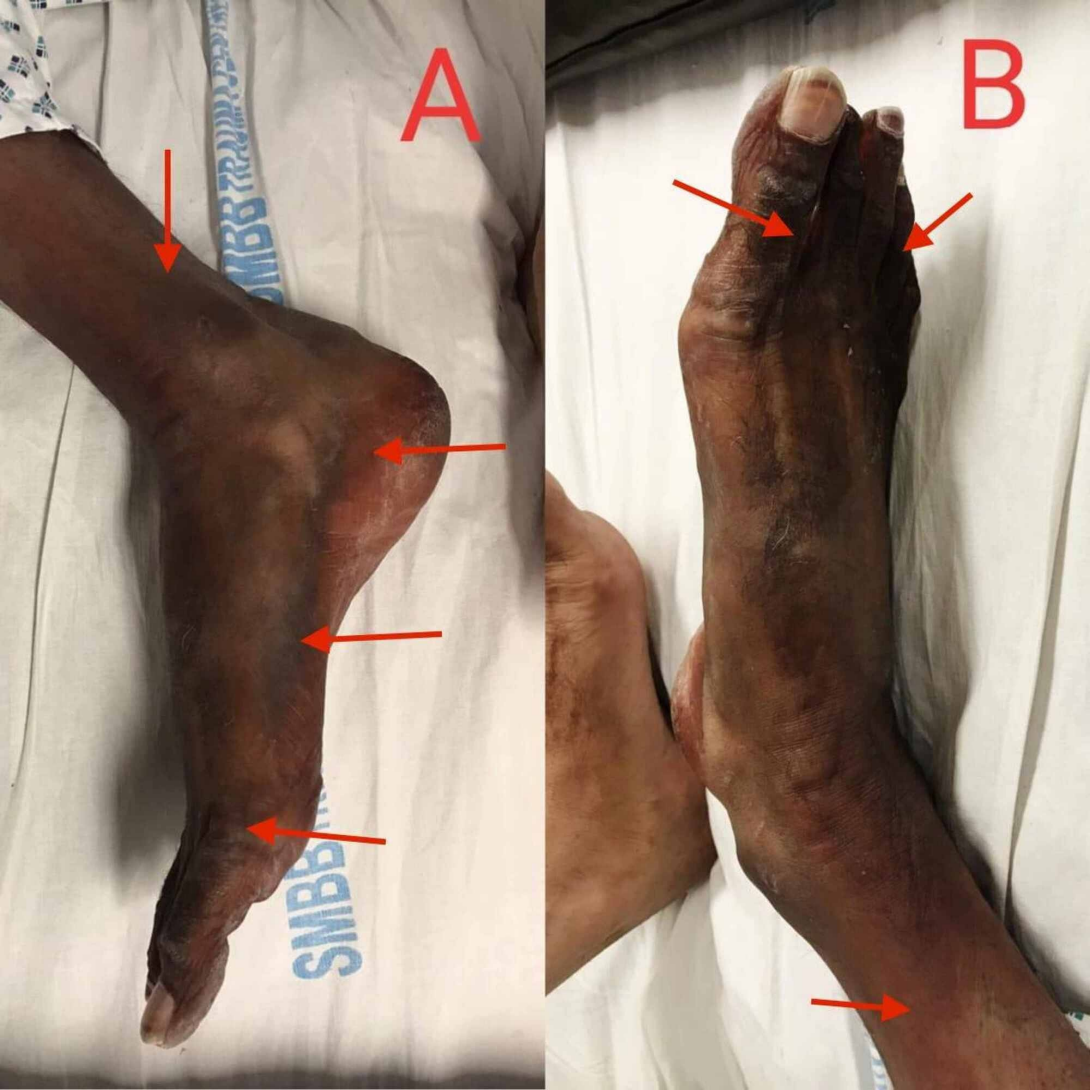
Ischemic limb of the patient The image shows the right lower limb (arrows indicating blackened ischemic areas) of the patient three days after admission.

On the third day of admission, the patient's oxygen requirements decreased. The WBC count increased to 45×10^9^/L, pO_2_ increased to 82 mmHg, and inflammatory markers were reduced. Chest X-ray also showed improvement. On the same day, the patient began complaining of pain in the right lower limb. A physical examination revealed hypothermia, numbness, and mild blackening of the right foot as shown in Figures 2A, 2B. Peripheral pulses of the right foot were not palpable. The vascular ultrasound of lower limbs showed no vascular flow in the popliteal, anterior tibial, posterior tibial, and dorsalis pedis artery. A severe arterial insufficiency was diagnosed, and the Vascular Surgery department was consulted immediately. They declared the limb unsalvageable and counselled for right limb above-knee amputation after the patient's condition had stabilized. Administering a therapeutic anticoagulant dose to prevent any other athero-embolic event was also suggested. Enoxaparin was then increased to 80 mg subcutaneous twice daily. Broad-spectrum antibiotics were added for the raised white cell count. On the ninth day of admission, the patient was maintaining oxygen saturation of 94% on room air. Chest X-ray showed a significant reduction in bilateral patchy infiltrates as shown in Figure 1B. The right foot was blackened and atrophied, which extended up to the knee. No progression in ischemia was noted. The patient was stepped down to high dependency unit (HDU) and was placed on the vascular surgery operating room list for above-knee amputation. The surgery took place under spinal anesthesia. After all aseptic measures, fish-mouth incision was given at the mid femur level through the skin, superficial fascia, and subcutaneous tissue. Deep tissue dissection and muscle transection was done. Neurovascular structures were identified, ligated, and transected. Lastly, the bone was lacerated, hemostasis was secured, and the wound was closed. Aseptic pressure dressing was applied, and the patient remained hemodynamically stable. Postoperatively, the patient was monitored in the HDU for 48 hours. The patient was then discharged and advised tablet rivaroxaban 10 mg once daily for one month.

## Discussion

COVID-19 is increasingly being associated with coagulation abnormalities leading to arterial and venous thromboses [5]. Klok et al. conducted a study on 184 patients with COVID-19, where the incidence of thrombosis was 31% overall, while 27% of cases were of venous thromboembolism and 3.7% showed arterial thrombotic events [9]. Our patient developed partial deep venous thrombosis and acute limb ischemia due to arterial thrombotic complications, after presenting with COVID-19 in a state of a CRS.

An arterial disease like acute limb ischemia is related to high mortality and morbidity rate [10]. The most common etiology of ALI is an arterial embolism, the majority of the emboli originating in the heart. However, our case presented with ALI owing to a viral infection, COVID-19. In most cases, if recognized early and managed with thrombolytics and interventions to provide early revascularization, the limb is salvaged [10]. However, ALI is associated with a high possibility of amputation if treatment is delayed. Our patient was treated with LMWH and TCZ since Day 1; however, he developed symptoms of ALI on Day 3, which worsened and led to blackening and atrophy of right leg extending up to the knee on Day 9. Once he was stable, the unsalvageable part of the ischemic limb was amputated; nevertheless, the patient attained stability.

Although hypercoagulability is associated with VTE more, COVID-19 patients show both venous and arterial manifestations [4]. The mechanism that predisposes to such condition can be explained by the association of COVID-19 with increased levels of pro-inflammatory cytokines (IL-2, IL-6, IL-7, G-CSF, TNF, IP-10, MCP1, MIP1-α, etc.) in patients with a severe disease, which leads to CRS [11,12]. Many studies suggest that severe COVID-19 infections cause increased levels of helper T lymphocytes, which excessively produce CD14 and CD16; these monocytes enhance the production of IL-6, which is a key mediator in COVID-19. IL-6 is a cytokine that triggers the production of acute-phase reactants (APRs), including CRP and fibrinogen from the liver. These two APRs play an important role in inducing the hypercoagulable state in the body and thus leading to thrombosis. Similarly, our patient presented with a markedly elevated CRP level (85 mg/L) exhibiting cytokine storm [8], which progressed to arterial thrombosis of the lower extremity. This leads to thrombosis, DIC, and eventual organ failure with raised D-dimer levels in patients, which is a marker of poor prognosis and higher mortality [3,13]. Likewise, our patient presented with elevated D-dimer levels along with coagulopathy that ultimately led to ischemia of his limb. However, prompt treatment with LMWH and TCZ resulted in a progressive decline in D-dimer levels and better prognosis. Another plausible pathogenesis of coagulation includes hypoxia. In conditions with severe COVID-19, hypoxia can trigger thrombosis by increasing hypoxia‐inducible transcription factor‐dependent signaling pathway as well as by increasing blood viscosity. Hence, early anticoagulation therapy was suggested to improve outcomes [7].

Heparin treatment is not only effective in managing coagulopathy, but its anti-inflammatory and anti-viral properties have also proven beneficial in COVID-19 patients. The intermediate dosage of heparin that is in between prophylactic and therapeutic doses might potentially reduce thrombotic events in COVID-19 patients [14]. Studies have shown that patients with elevated D-dimers are shown to benefit from anticoagulation therapy, specifically LMWH [7].

On the other hand, IL-6 level monitoring is an essential step in recognizing the development of COVID-19 complications and planning its treatment accordingly. Clinical trials of TCZ have achieved a triumph in treating COVID-19 patients worldwide [8]. A patient on TCZ therapy develops a sudden rise and fall in levels of IL-6. This rise can be explained by the mechanism of TCZ, which binds to and inhibits the IL-6 receptor, downregulating the clearance of IL-6 and enhancing its accumulation in serum. However, the levels gradually decrease due to effective treatment with TCZ, which turns down the cytokine storm [15]. Hence, our patient received co-management of LMWH and TCZ, which showed promising results and exhibited mortality benefit. It subsided the cytokine storm, halting the progression of ischemia.

## Conclusions

Although ALI is a rare and severe complication of COVID-19, its outcomes can be improved by timely diagnosis and effective medical and surgical interventions. Hence, we concluded that heparin is effective in treating CAC as well as thrombotic events, while TCZ, simultaneously, decreases the severity of CRS. This might reduce the severity of COVID-19-induced complications synergistically. The timely use of TCZ along with anticoagulation therapy in the proper dosage might show a beneficial outcome in CAC. Further research on this would be a breakthrough during these times and would aid in the proper management of the complication and might decrease the mortality rate owing to COVID-19.
